# An elevated glycemic gap predicts adverse outcomes in diabetic patients with necrotizing fasciitis

**DOI:** 10.1371/journal.pone.0223126

**Published:** 2019-10-03

**Authors:** Po-Chuan Chen, Shih-Hung Tsai, Jen-Chun Wang, Yuan-Sheng Tzeng, Yung-Chih Wang, Chi-Ming Chu, Shi-Jye Chu, Wen-I Liao

**Affiliations:** 1 Department of Emergency Medicine, Tri-Service General Hospital, National Defense Medical Center, Taipei, Taiwan; 2 Department of Physiology and Biophysics, Graduate Institute of Physiology, National Defense Medical Center, Taipei, Taiwan; 3 Division of Plastic and Reconstructive Surgery, Department of Surgery, Tri-Service General Hospital, National Defense Medical Center, Taipei, Taiwan; 4 Division of Infectious Diseases and Tropical Medicine, Department of Internal Medicine, Tri-Service General Hospital, National Defense Medical Center, Taipei, Taiwan; 5 School of Public Health, National Defense Medical Center, Taipei, Taiwan; 6 Department of Internal Medicine, Tri-Service General Hospital, National Defense Medical Center, Taipei, Taiwan; Wayne State University, UNITED STATES

## Abstract

**Background:**

Diabetes is the most common comorbidity of necrotizing fasciitis (NF), but the effect of stress-induced hyperglycemia (SIH) on diabetic patients with NF has never been investigated. The aim of this study was to assess whether SIH, as determined by the glycemic gap between admission glucose levels and A_1C_-derived average glucose levels, predicts adverse outcomes in diabetic patients hospitalized with NF.

**Methods:**

We retrospectively reviewed the glycemic gap and clinical outcomes in 252 diabetic patients hospitalized due to NF from 2011 to 2018 in a single medical center in Taiwan. A receiver operating characteristic (ROC) curve was used to analyze the optimal cutoff values for predicting adverse outcomes. Univariate and multivariate logistic regression analyses were employed to identify significant predictors of adverse outcomes.

**Results:**

In total, 194 diabetic NF patients were enrolled. Compared with patients without adverse outcomes, patients with adverse outcomes had significantly higher glycemic gaps, Acute Physiology and Chronic Health Evaluation (APACHE) II scores and C-reactive protein (CRP) levels; lower albumin and hemoglobin levels; greater incidence of limb loss; and longer hospital and intensive care unit stays. The glycemic gap positively correlates with the laboratory risk indicator for NF scores, APACHE II scores and CRP levels. A glycemic gap of 146 mg/dL was the optimal cutoff value for predicting adverse outcomes using the ROC curve. Compared with patients with glycemic gaps ≤146 mg/dL, those with glycemic gaps >146 mg/dL had higher APACHE II scores and incidence rates of adverse outcomes, especially bacteremia and acute kidney injury. Multivariate analysis revealed that a glycemic gap >146 mg/dL and APACHE II score >15 were independent predictors of adverse outcomes, while the presence of hyperglycemia at admission was not.

**Conclusions:**

An elevated glycemic gap was significantly independently associated with adverse outcomes in diabetic NF patients. Further prospective studies are warranted to validate the role of the glycemic gap in NF patients with diabetes.

## Introduction

Necrotizing fasciitis (NF) is a rapidly progressing soft-tissue infection that leads to adverse outcomes, including systemic toxicity, septic shock and death. The mortality rate could be as high as 7.6~40.6% if adequate treatment is not administered [1–3]. Patients with a compromised immune system are more likely to develop NF. Diabetes is the most important comorbidity of NF, accounting for 52.1% to 70.8% of the overall population of patients with NF [3–5]. Compared with nondiabetic patients with NF, diabetic patients with NF have higher rates of amputation and polymicrobial infections and worse outcomes [6–8]. To improve survival and limit limb loss, early diagnosis, aggressive debridement of necrotic fascia and subcutaneous tissue and the early administration of antimicrobial therapy remains the main treatment strategy.

Many critical illnesses cause stress-induced hyperglycemia (SIH) via the production of excessive catecholamine, glycogen and inflammatory cytokines, resulting in increased gluconeogenesis and insulin resistance [9]. SIH could be a hallmark of disease severity in patients with acute illnesses and is independently associated with adverse outcomes in patients with many different critical conditions, such as community-acquired pneumonia, sepsis, burn injuries, major trauma and myocardial infarction [10–13]. However, there is still some controversy regarding the differences in SIH between diabetic and nondiabetic patients. Egi et al. demonstrated that background hyperglycemia may affect the interaction between acute hyperglycemia and mortality in diabetic patients with critical illnesses [14]. In addition, the association between admission hyperglycemia and adverse outcomes in infected diabetic patients is still debated. Schuetz et al. found that admission hyperglycemia was associated with adverse outcomes in a nondiabetic acutely infected population but not in diabetic patients [15]. Similar associations were observed in patients with other infections, such as severe sepsis, community-acquired pneumonia and surgical-site infections [16–19]. Indeed, admission hyperglycemia in acutely ill diabetic patients could be divided into the following two categories: SIH resulting from acute physiological stress and chronically elevated baseline blood glucose levels. The conflicting findings may be partly attributed to the fact that the hyperglycemic state may result from long-standing hyperglycemia rather than actual SIH in diabetic patients with infectious critical illnesses.

Glycated hemoglobin (HbA_1c_) is relatively unaffected by acute stress or sepsis and can represent the level of glycemic control over the preceding 2–3 months [20]. The A1C-Derived Average Glucose (ADAG) study included 643 participants and found a validated correlation between HbA1c levels and long-term mean plasma glucose levels, allowing the translation of HbA_1c_ values to long-term average glucose levels [20]. To evaluate the real effect of SIH, we used the glycemic gap (the ADAG subtracted from the admission glucose level) to eliminate the interference of chronically elevated baseline blood glucose levels in diabetic patients with various critical illnesses. Our studies and other studies have found that the glycemic gap is associated with disease severity and adverse clinical outcomes in diabetic patients with liver abscesses, community-acquired pneumonia, acute myocardial infarction, acute ischemic stroke, chronic obstructive lung disease, trauma and in-hospital cardiac arrest [21–27].

The significance of hyperglycemia or the glycemic gap in diabetic patients with NF has yet to be clarified. We hypothesized that a larger glycemic gap rather than an elevated admission glucose level may be associated with adverse outcomes in patients with diabetes and NF. The purpose of the present study was to further investigate if an elevated glycemic gap can be used to predict adverse clinical outcomes in diabetic patients with NF. In addition, the relationships between clinical data, microbiology and clinical outcomes were analyzed.

## Materials and methods

### Patient selection

The institutional review board for human investigations of Tri-Service General Hospital, a tertiary referral medical center in northern Taiwan, approved this retrospective study and waived the need to obtain informed consent because the study was retrospective and the medical records were deidentified. In this observational study, all patients with diabetes admitted for NF between January 1, 2011, and October 31, 2018, were identified by searching for the 9^th^ revision International Classification of Diseases (ICD) codes 728.86 and 250.2–8 plus the 10^th^ revision ICD codes M72.6 and E10-14. The data associated with the identified patients were then reviewed, and only those patients who had plasma glucose levels measured at their initial presentation were selected. HbA1c level data were determined in patients within one month before or one week after their admission. Patients were excluded if they had concurrent infections, used steroids, lacked data on HbA1c levels within the prescribed time limit or presented with hypoglycemia upon admission (plasma glucose < 70 mg/dl). To avoid confounding effects, we also excluded individuals with medical conditions that could alter the process of hemoglobin glycation, such as hemoglobinopathies (e.g., sickle cell anemia, thalassemia) or hematologic conditions (e.g., hemolytic anemia), and a history of splenectomy.

### Study design

We retrospectively reviewed the medical records of NF patients with diabetes to determine age, sex, underlying comorbidities (including uremia, peripheral arterial occlusive disease, liver cirrhosis, cancer, alcoholism and drug abuse), clinical presentation, time from diagnosis to surgery, Acute Physiology and Chronic Health Evaluation (APACHE) II score at admission, HbA_1c_ levels_,_ laboratory data, laboratory risk indicator for NF (LRINEC) score, blood and wound culture results, adverse outcomes, number of times wound debridement was performed, limb amputation status, and lengths of stay in the intensive care unit (ICU) and the hospital. The definitive diagnosis of NF depended on surgical findings that included the lack of resistance of adherent fascia to digital blunt dissection, necrotic fascia and a purulent discharge with a foul “dish water” odor [4]. Diabetes was confirmed with an HbA1c level >6.5% in the preceding 2 months [28] and/or if the patient was discharged with the diagnosis of either type 1 or type 2 diabetes and/or treated with insulin or oral antidiabetic agents. All patients were treated with broad-spectrum antibiotic therapy, aggressive radical debridement and adequate resuscitation. All wound cultures were obtained from the necrotic tissue during the first operation. Monomicrobial infection was defined as a single species of bacteria isolated from the wound culture or blood culture collected in the emergency department before the administration of antibiotic therapy. The adverse outcomes were as follows: (1) acute kidney injury (AKI; defined as serum creatinine elevated > 0.5 mg/dL from the baseline, which was recorded when hemoglobin A1C was measured prior to admission or on admission); (2) acute respiratory failure that required endotracheal tube intubation and mechanical ventilation support; (3) bacteremia (defined as a positive blood culture); (4) septic shock (defined by the clinical criteria of sepsis and vasopressor therapy needed to maintain a mean arterial pressure of 65 mm Hg and lactate >2 mmol/L in the absence of hypovolemia) [29]; and (5) mortality during admission. The anatomical site of infection was classified as either central (head, neck, trunk and perineum) or peripheral (upper and lower extremities) [5]. Limb loss was defined as amputation above the ankle or the wrist [8].

#### Measurements of plasma glucose levels, HbA_1c_ levels and glycemic gaps

The admission plasma glucose level was defined as the level determined when the patient initially presented to the emergency department. HbA_1C_ assays were performed with a blood analyzer (Primus CLC 385; Primus Corporation, Kansas City, MO, USA) equipped with a high-performance liquid chromatography system at Tri-Service General Hospital (Taipei, Republic of China). The laboratory received Level 1 certification for this analysis from the National Glycohemoglobin Standardization Program.

We use the following equation to convert the HbA1c level to the estimated long-term average glucose level (eAG) over the previous 3 months: eAG = 28.7 × HbA_1c_ − 46.7 [20]. The glycemic gap was calculated as the admission glucose levels obtained at the time of initial presentation minus the eAG level. The stress hyperglycemia ratio (SHR) was calculated from the blood glucose level at admission divided by the eAG level.

#### Statistical analysis

Normally distributed continuous data are expressed as the means ± standard deviations and were analyzed with two-tailed Student’s *t*-tests. Nonnormally distributed continuous data were analyzed with the Mann-Whitney U test and are presented as the median (25%-75% interquartile range). Categorical data are presented as frequencies (%) and were analyzed using the chi-square test or Fisher’s exact test. The rate of limb loss was calculated using the peripheral location number as the denominator. One-way analysis of variance was used to evaluate the significance of various characteristics, laboratory data, and adverse outcomes. The multitudinous comparisons between groups were adjusted with Bonferroni post hoc tests. The correlations between glycemic gap levels and C-reactive protein (CRP) levels, APACHE II scores or LRINEC scores were determined with Pearson correlation. We also plotted a receiver operator characteristic (ROC) curve to classify the adverse outcomes in diabetic NF patients on the basis of the glycemic gap. The DeLong method was used to compare the area under the curve (AUC) values from the ROC analysis. Univariate logistic regression analysis was performed for all possible predictors associated with adverse outcomes. The identified variables for which the *P*-value <0.05 in the univariate analysis plus age and sex were further assessed with the multivariate analysis to determine the predictors most strongly associated with adverse outcomes. We estimated the final logistic regression model with ROC curves. The data were analyzed with Statistical Package for the Social Sciences version 22.0 (SPSS, Inc., Chicago, IL, USA). Differences with *P*-values <0.05 were considered statistically significant.

## Results

### Demographic data and underlying disease

In total, 252 patients with the admission diagnoses of NF and diabetes were initially identified; 7 were excluded because they had hypoglycemia on admission, and 51 were excluded because they lacked HbA1c level and glucose data on admission. No patients were excluded for hemoglobinopathies, hematologic conditions or history of splenectomy in this study. We enrolled 194 patients after a subsequent chart review. The demographic data and comparisons of clinical features of the enrolled patients are shown in Table 1. There were no statistically significant differences in comorbidities between diabetic NF patients with and without adverse outcomes. Among the 194 patients with NF, the lower extremities were involved in 162 patients (83.5%), the upper extremities were involved in 6 (3.1%), and the head, neck, trunk and perineum (central location) were involved in 26 (13.4%). Compared with patients without adverse outcomes, patients with adverse outcomes had a significantly higher incidence of upper limb NF (*P* = 0.01) but a lower incidence of lower limb NF (*P* = 0.03).

**Table 1:** The comparison of the characteristics and underlying diseases in diabetic patients with necrotizing fasciitis.

	All patients (n = 194)	Patients without adverse outcomes (n = 124)	Patients with adverse outcomes (n = 70)	*P*-value
Age (years)	59.5 ± 12.3	60.4 ± 11.4	59.1 ± 12.9	0.48
Male	125 (64.4%)	82 (66.1%)	43 (61.4%)	0.51
***Underlying disease***				
Uremia	38 (19.6%)	27 (21.8%)	11 (15.7%)	0.31
PAOD	38 (19.6%)	23 (18.5%)	15 (21.4%)	0.63
Liver cirrhosis	10 (5.2%)	6 (4.8%)	4 (5.7%)	0.79
Cancer	8 (4.1%)	4 (3.2%)	4 (5.7%)	0.40
Alcoholism	3 (1.5%)	2 (1.6%)	1 (1.4%)	0.92
Drug abuse	3 (1.5%)	2 (1.6%)	1 (1.4%)	0.92
***Location***				
Peripheral	168 (86.6%)	110 (88.7%)	58 (82.8%)	0.25
Upper limb	6 (3.1%)	1 (0.8%)	5 (7.1%)	0.01*
Lower limb	162 (83.5%)	109 (87.9%)	53 (75.7%)	0.03*
Central	26 (13.4%)	14 (11.3%)	12 (17.6%)	0.25
Time from diagnosis to surgery (hrs)[IQR]	12.9[4.9–20.7]	12.0[4.3–20.5]	14.3[5.8–23.8]	0.15

PAOD, peripheral arterial occlusive disease; IQR, interquartile range; Central location defined as head, neck, trunk and perineum; Values are expressed as the mean± SD, number (%) or mean [25%-75% IQR]

*P<0.05

### Laboratory data and NF-related parameters

As shown in Table 2, compared with diabetic NF patients without adverse outcomes, those with adverse outcomes had a significantly larger glycemic gap (*P* = 0.02); higher SHR (defined by the blood glucose level at admission divided by the eAG level, *P* = 0.01), APACHE II score (*P*<0.001), and CRP (*P* = 0.003) level; lower albumin (*P*<0.001) and hemoglobin (*P* = 0.04) levels; greater incidence of limb loss (*P* = 0.01); and longer ICU (*P*<0.001) and hospital (*P* = 0.004) stays. However, there were no significant differences in HbA1c values or admission glucose levels between patients with and without adverse outcomes.

**Table 2:** Results of investigations performed at the time of initial presentation and necrotizing fasciitis-related parameters.

	All patients (n = 194)	Patients without adverse outcomes (n = 124)	Patients with adverse outcomes (n = 70)	*P*-value
Glycemic gap, mg/dL[IQR]	76.4[3.3–143.2]	58.0 [-15.6–124.2]	109.1[8.8–202]	0.02*
Stress hyperglycemia ratio[IQR]	1.37[1.0–1.6]	1.27[0.9–1.5]	1.54[1.1–1.9]	0.01*
HbA1c, %[IQR]	9.3[7.0–11.4]	9.5[7.2–11.4]	9.0[6.8–11.1]	0.17
Glucose, mg/dL[IQR]	297.7[176.8–388.0]	284.7[168.8–368.8]	320.8[181.3–414.3]	0.17
White cell count, μL	16186.0 ± 7414.9	15797.4 ± 7033.3	16874.4 ± 8053.1	0.33
Hemoglobin, g/dL	11.2 ± 2.3	11.8 ± 2.2	10.8 ± 2.4	0.04*
CRP, mg/dL[IQR]	19.6[11.5–27.0]	17.7[9.3–25.4]	22.8[14.1–32.3]	0.003*
Na, mmol/L	130.9 ± 5.6	130.9 ± 4.9	130.8 ± 6.7	0.85
Cr, mg/dL[IQR]	2.4[0.9–2.7]	2.5[0.9–2.6]	2.2[0.9–2.8]	0.42
Albumin, mg/dL	2.7 ± 0.6	2.9 ± 0.6	2.4 ± 0.6	<0.001*
APACHE II score[IQR]	11.8[6.0–15.0]	9.9[6.0–13.8]	14.7[9.0–18.3]	<0.001*
LRINEC score (%)				
Mean (range)	7.5 ± 3.0	7.2 ± 3.0	8.0 ± 2.9	0.08
≤ 6	62 (32.0%)	44 (35.5%)	18 (25.7%)	0.16
> 8	99 (51.0%)	58 (46.8%)	41 (58.6%)	0.11
Polymicrobial	92 (47.4%)	64 (51.6%)	28 (40.0%)	0.12
Monomicrobial KP	10 (5.2%)	4 (3.2%)	6 (8.6%)	0.11
Monomicrobial SA	23 (11.9%)	13 (10.5%)	10 (14.3%)	0.43
MRSA	7 (3.6%)	3 (2.4%)	4 (5.7%)	0.24
MSSA	16 (8.3%)	10 (8.1%)	6 (8.6%)	0.90
ICU admission	54 (27.8%)	8 (6.5%)	46 (65.7%)	<0.001*
ICU stay, days[IQR]	5.2[0–3]	0.3[0–0]	13.7[0–19.3]	<0.001*
Hospital stay, days[IQR]	34.0[18.0–41.3]	29.3[18.0–37.0]	42.3[22.8–48.8]	0.004*
No. of debridements	4.1 ± 3.5	4.0 ± 3.3	4.3 ± 3.9	0.62
STSG	113 (58.2%)	68 (55.7%)	45 (64.3%)	0.25
Limb loss^#^	34 (20.2%)	16 (14.5%)	18 (31.0%)	0.01*

IQR, interquartile range; CRP, C-reactive protein; Cr, creatinine; APACHE, acute physiologic and chronic health evaluation; LRINEC, laboratory risk indicator for necrotizing fasciitis; KP, *Klebsiella pneumoniae*; SA, *Staphylococcus aureus*; MRSA, methicillin-resistant *Staphylococcus aureus*; MSSA, methicillin-sensitive *Staphylococcus aureus*; ICU, intensive care unit; No., number; STSG, split-thickness skin graft; Limb loss, defined as amputation above the ankle or the wrist; Values are expressed as the mean± SD, number (%) or mean [25%-75% IQR]

*P<0.05

^#^ The rate of limb loss was calculated using the peripheral location number as the denominator.

**Fig 1:**
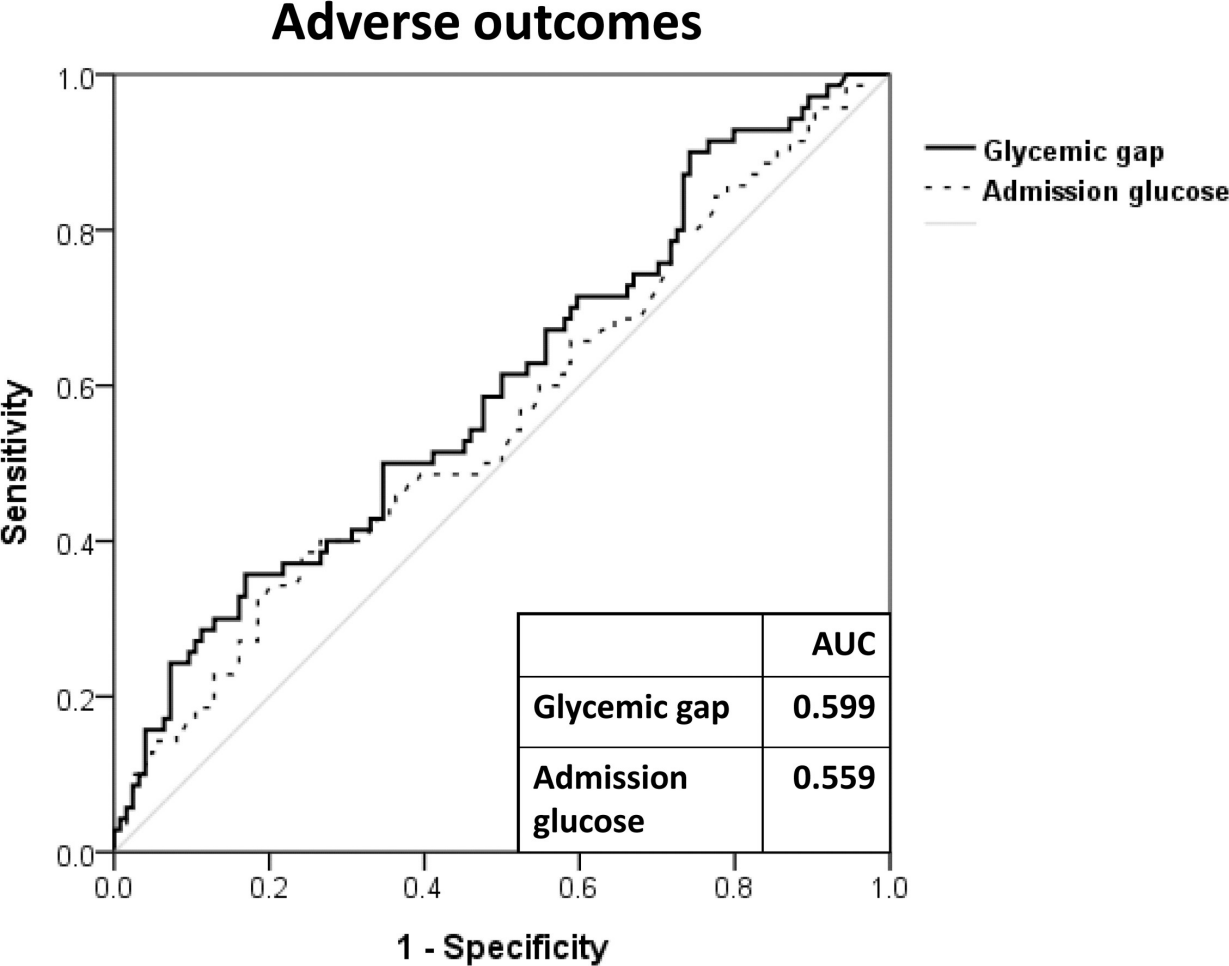
ROC analysis of glycemic gaps and glucose levels at admission for the prediction of adverse outcomes among diabetic NF patients. AUC; Area under the curve; ROC; Receiver operating characteristic.

**Fig 2:**
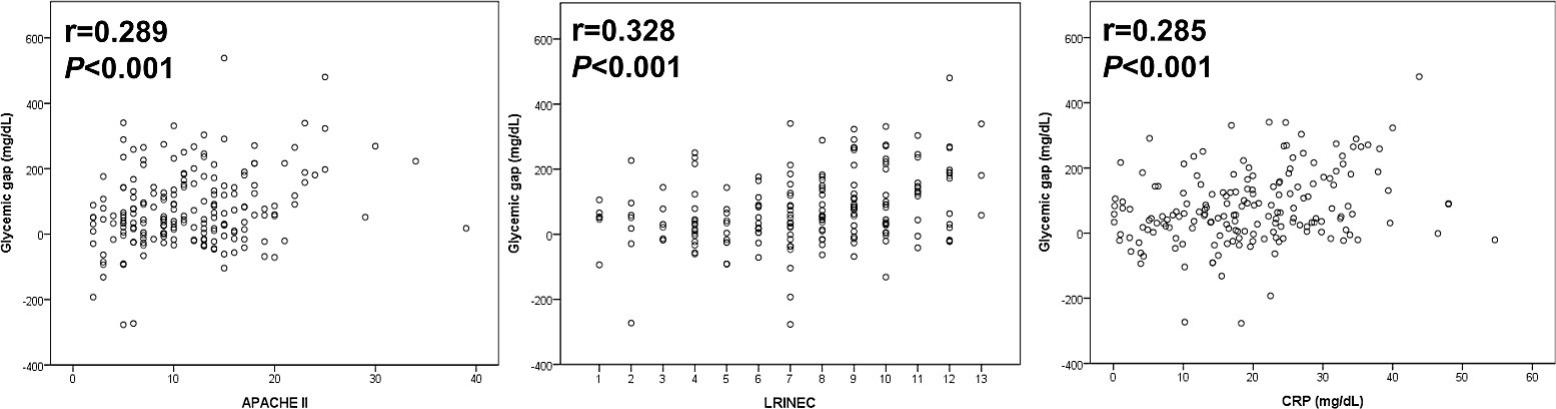
Correlations between the glycemic gap and the APACHE II scores, LRINEC scores and CRP levels.

Compared with admission glucose levels [0.559, 95% confidence interval (CI): 0.474–0.645], glycemic gaps showed greater AUC values (0.599, 95% CI: 0.515–0.683) for predicting adverse outcomes (Fig 1). However, the difference between the glycemic gap and admission glucose levels (*P* = 0.12) was not significant. In addition, SHR produced higher AUC values (0.608, 95%CI: 0.535–0.677) than did glycemic gap for predicting adverse outcomes, but this difference was also not significant (*P* = 0.40). As shown in Fig 2, there were statistically significant positive correlations between the glycemic gap and the APACHE II scores (r = 0.289, *P*<0.001), LRINEC scores (r = 0.328, *P*<0.001) and CRP levels (r = 0.285, *P*<0.001).

### The glycemic gap and adverse outcomes

We used the Youden index to determine an optimal cutoff value of 146 mg/dL, with sensitivity and specificity of 35.7% and 83.1%, respectively, for the development of adverse outcomes. Compared with diabetic NF patients with a glycemic gap≤146 mg/dL (30.4%), patients with glycemic gaps >146 mg/dL (54.3%) had a 23.9% relative increase in the incidence of combined adverse outcomes (*P* = 0.003), especially bacteremia (*P* = 0.006) and AKI (*P* = 0.04) (Table 3). Further analysis showed that patients with a glycemic gap >146 mg/dL had higher APACHE II score (*P*<0.001) and a significantly elevated risk of monomicrobial *Klebsiella pneumoniae* (KP) infection (*P* = 0.04).

**Table 3:** Clinical outcomes in diabetic patients with necrotizing fasciitis stratified by the glycemic gap.

	Glycemic gap≤146 mg/dL (n = 148)	Glycemic gap>146 mg/dL (n = 46)	*P*-value
Adverse outcomes	45 (30.4%)	25 (54.3%)	0.003*
Mortality	8 (5.4%)	4 (8.7%)	0.42
Septic shock	19 (12.8%)	7 (15.2%)	0.68
Bacteremia	19 (12.8%)	14 (30.4%)	0.006*
Acute respiratory failure	29 (19.6%)	9 (19.6%)	0.99
Acute kidney injury	22 (14.9%)	13 (28.3%)	0.04*
Polymicrobial	72 (48.6%)	20 (43.5%)	0.54
Monomicrobial KP	5 (3.4%)	5 (10.9%)	0.04*
Monomicrobial SA	14 (9.5%)	9 (19.6%)	0.06
MRSA	4 (2.7%)	3 (6.5%)	0.23
MSSA	10 (6.8%)	6 (13.0%)	0.18
NO. of debridements	4.1 ± 3.7	4.1 ± 3.1	0.95
STSG	92 (62.2%)	22 (47.8%)	0.08
Limb loss^#^	23 (17.6%)	11 (29.7%)	0.10
APACHE II score[IQR]	10.6[6.0–14.0]	14.7[10.0–18.8]	<0.001*

KP, *Klebsiella pneumoniae*; SA, *Staphylococcus aureus*; MRSA, methicillin-resistant *Staphylococcus aureus*; MSSA, methicillin-sensitive *Staphylococcus aureus*; NO., number; STSG, split-thickness skin graft; APACHE, acute physiologic and chronic health evaluation; IQR, interquartile range; Limb loss, defined as amputation above the ankle or the wrist; Values are expressed as the mean± SD, number (%) or mean [25%-75% IQR]

*P<0.05

^#^ The rate of limb loss was calculated using the peripheral location number as the denominator.

### Univariate and multivariate logistic regression analysis

Univariate analysis showed that glycemic gaps >146 mg/dL, APACHE II score>15 and creatinine levels >1.6 mg/dL were correlated with adverse outcomes (*P* <0.05), but admission glucose levels >180 mg/dL were not similarly correlated (Table 4).

**Table 4:** Univariate logistic regression analysis of risk factors associated with adverse outcomes.

Variable	Univariate analysis Odds ratio (95%CI)
Age > 65 years	1.1 (0.6–2.0)
Male	1.2 (0.7–2.3)
Uremia	1.5 (0.7–3.2)
PAOD	1.3 (0.6–2.8)
Liver cirrhosis	1.2 (0.3–4.4)
Cancer	1.8 (0.4–7.5)
Glycemic gap >146 mg/dL	2.7 (1.4–5.4)*
WBC >15000/μL	0.9 (0.5–1.6)
Hb <11 g/dL	1.4 (0.8–2.6)
Cr >1.6 mg/dL	1.9 (1.1–3.5)*
Sodium <135 mmol/L	1.3 (0.7–2.5)
Glucose >180 mg/dL	1.2 (0.6–2.4)
CRP >15 mg/dL	1.5 (0.8–2.9)
Albumin <3.5 mg/dL	7.5 (0.9–60.0)
LRINEC score >8	1.6 (0.9–2.9)
APACHE II score >15	3.7(1.8–7.4)*
Central location	1.6 (0.7–3.7)

CI, confidence interval; PAOD, peripheral arterial occlusive disease; WBC, white cell count; Cr, creatinine; CRP, C-reactive protein; LRINEC, laboratory risk indicator for necrotizing fasciitis; APACHE, acute physiologic and chronic health evaluation

*P<0.05

We selected all parameters for which the *P*-value was <0.05 in the initial univariate results (glycemic gaps >146 mg/dL, creatinine levels >1.6 mg/dL and APACHE II score>15), age >65 years and male sex for inclusion in the multivariate analysis. Multivariate analysis showed that a glycemic gap >146 mg/dL [Odds ratio (OR): 2.4, 95% CI: 1.2–4.9, *P* = 0.02] and APACHE II score>15 (OR: 3.1, 95% CI: 1.3–7.1, *P* = 0.01) were independent predictors in the incidence of adverse outcomes in diabetic NF patients (Table 5). The final logistic regression model had an AUC value of 0.685 (95% CI: 0.606–0.764, *P*<0.001).

**Table 5:** Multivariate logistic regression analysis of risk factors associated with adverse outcomes.

Variable	Multivariate analysis
	Odds ratio (95%CI)
Age > 65 years	1.1 (0.6–2.2)
Male	1.0 (0.5–2.0)
Glycemic gap >146 mg/dL	2.4 (1.2–4.9)*
APACHE II score>15	3.1 (1.3–7.1)*
Cr >1.6 mg/dL	1.2 (0.6–2.4)

CI, confidence interval; APACHE, acute physiologic and chronic health evaluation; Cr, creatinine; Multivariate analysis included all variables with *P* values <0.05 in the univariate analysis plus age >65 years and male sex

*P<0.05

### Microbiology

Wound and blood cultures were obtained for all patients. There were 166 (85.6%) positive wound cultures and 33 (17%) positive blood cultures (Table 6). Among the patients with positive wound cultures, 92 (47.4%) patients had polymicrobial cultures, and the most common gram-positive microorganism was SA (27.7%, n = 46), followed by *Streptococci pyogenes (*A or other *b-hemolytic strains*) (23.4%, n = 39). The most common gram-negative microorganism was KP (14.5%, n = 24). Moreover, SA was the most common pathogen leading to bacteremia (36.4%, n = 12), particularly MRSA (24.2%, n = 8), followed by KP (21.2%, n = 7).

**Table 6:** Pathogenic microorganisms isolated from patients with necrotizing fasciitis.

Microbiology	Positive wound culture (n = 166)	%	Positive blood culture (n = 33)	%
**Gram-positive bacteria**				
*Staphylococcus aureus*	46	27.7	12	36.4
*MSSA*	28	16.9	4	12.1
*MRSA*	18	10.8	8	24.2
*Enterococcus faecalis*	26	15.7	1	3
*Streptococcus spp*.	9	5.4	0	0
*B-Streptococcus group A*	4	2.4	1	3
*B-Streptococcus group B*	19	11.4	0	2.6
*B-Streptococcus non-A*,*B*,*D*	7	4.2	0	0
*CoNS*	7	4.2	2	6
*Viridans streptococci*	12	7.2	1	3
Others	9	5.4	3	9
**Gram-negative bacteria**				
*Klebsiella pneumoniae*	24	14.5	7	21.2
*Escherichia coli*	13	7.8	3	9
*Proteus spp*.	20	12	2	6
*Pseudomonas aeruginosa*	6	3.6	1	3
*Prevotella spp*.	16	9.6	0	0
*Bact*. *thetaiotaomicron*	4	2.4	1	3
*Veillonella parvula*	4	2.4	0	0
*Enterobacter cloacae*	6	3.6	0	0
*Morganella morganii*	7	4.2	1	3
*Serratia marcescens*	4	2.4	0	0
*Citrobacter spp*.	6	3.6	0	0
Other	10	6	1	3
**Anaerobic bacteria**				
*Bacteroides spp*.	38	22.9	2	6
*Peptostreptococcus spp*.	3	1.8	0	0
*Others*				
*Clostridium spp*.	1	0.6	0	0
*Yeast*	3	1.8	0	0

MSSA, *methicillin-sensitive Staphylococcus aureus;* MRSA, *methicillin-resistant Staphylococcus aureus*; CoNS, *coagulase-negative Staphylococcus*

## Discussion

This study is the first to investigate if an elevated glycemic gap can be used to predict adverse clinical outcomes in diabetic patients with NF. Our main findings are as follows: 1). An elevated glycemic gap and not hyperglycemia at admission or poor chronic glycemic control was related to adverse outcomes in diabetic patients with NF. 2). A glycemic gap>146 mg/dL and APACHE II score>15 independently predicted the development of adverse outcomes, especially bacteremia and AKI, after adjusting for typical NF risk factors.

Diabetic patients have increased susceptibility to infection, and SIH has been reported to be correlated with adverse outcomes in patients with various critical illnesses and infectious diseases [10, 11, 30]. On the other hand, some investigators observed a protective effect of acute hyperglycemia in patients with diabetes and certain critical and infectious conditions [31, 32], although the underlying mechanisms have not been elucidated. It has been suggested that SIH may have distinct pathophysiologic effects in nondiabetic and diabetic patients. In a recent large study with 41492 critically ill patients with sepsis, hyperglycemia at admission was associated with elevated mortality rates in nondiabetic patients but not in diabetic patients [18]. In addition, hyperglycemia at admission was significantly independently associated with surgical-site infections in nondiabetic orthopedic trauma patients [19]. The different ability of hyperglycemia at admission to predict adverse outcomes in critically ill and infected patients with and without diabetes may be the result of the different baseline glucose levels in the two groups of patients. Because patients with diabetes have various levels of chronic glycemic control, it is necessary to consider background hyperglycemia when investigating the association between SIH and adverse outcomes in diabetic patients.

Regarding the previous studies, there are limited data focused on the relationships among hyperglycemia at admission, chronic glycemic control level and clinical outcomes in diabetic patients with NF. Some studies found that the admission glucose level was not different between nonsurviving and surviving NF patients [33, 34]. In a small series of 15 diabetic NF patients, the mean blood glucose levels and HbA1c levels were higher in the nonsurviving group than in the surviving group [35]. Our study demonstrated that the glycemic gap, which eliminated the effect of chronic glycemic levels, reflects physiological stress in diabetic NF patients more accurately than does a single plasma glucose value at admission. In addition to the glycemic gap, Roberts et al. demonstrated that the SHR, calculated as the blood glucose level at admission divided by the estimated average glucose level, can eliminate the interference of background glycemic levels and served as a better predictor than acute hyperglycemia of critical illness [36]. Another similar method, the “Hemoglobin Glycation Index”(HGI), which is calculated as the measured HbA1c level minus the HbA1c level estimated from the fasting glucose level, was associated with cardiovascular disease in patients with impaired glucose metabolism [37]. In our previous study, both the glycemic gap and SHR were useful in assessing the disease severity of patients presenting with acute ischemic stroke [24]. In the current study, we disclosed similar findings to those of the acute ischemic stroke study, and the difference was not significant between the glycemic gap and SHR methods. We believe that the glycemic gap and SHR may provide new insight into the paradox of discordant results regarding the relationships between hyperglycemia at admission and adverse outcomes in NF patients with preexisting diabetes.

Bacteremia is one of the five most common complications of NF [34], and it is associated with a higher mortality rate in diabetic patients than in nondiabetic patients [4, 38]. Cheng et al. found that bacteremia is a significant predictor of death in diabetic NF patients, which is consistent with our result [8]. Patients with an elevated glycemic gap had a significantly higher incidence of bacteremia, which may be representative of elevated levels of inflammatory cytokines in response to bacterial endotoxins or exotoxins. NF patients with diabetes often have polymicrobial infections and monomicrobial KP infections [7, 8]. In the current study, among the monomicrobial infections, the most common causative gram-positive microorganism was *Staphylococcus aureus* (SA), and the most common gram-negative causative microorganism was KP, which is in accordance with the findings of previous studies [4]. Sepsis-induced microvascular dysfunction, a decreased glomerular filtration rate and a vigorous inflammatory response were factors substantially affecting the development of AKI. A previous study found that AKI was the most common complication (36%) in NF patients [34]. In addition, Uehara et al. demonstrated that the development of AKI increased the risk of mortality 15-fold in patients with NF [39]. In this study, patients with a glycemic gap >146 mg/dL had an incidence of AKI approximately twice that of patients with a glycemic gap ≤146 mg/dL, which represents an elevated glycemic gap associated with a more severe septic response.

The APACHE II score, a disease severity scoring system, has been applied as a prediction tool for risk evaluation in critically ill patients. A higher APACHE II score corresponds to more severe disease and a higher risk of mortality. A previous study conducted by Liu et al. proposed that an APACHE II score>15 was independently associated with an unfavorable outcome in patients with sepsis [40]. Our study also found that an APACHE II score>15 predicted adverse outcomes in diabetic patients with NF. Furthermore, the glycemic gap has weak positive correlations with APACHE II scores, LRINEC scores and CRP levels, implying that an elevated glycemic gap may reflect greater severity of illness and physiological stress leading to the development of adverse outcomes.

### Limitations

Our study had several limitations. First, this retrospective study lacked information regarding the exact time antibiotic therapy was first administered and the time from onset of symptoms to admission to the hospital, which may interfere with the analysis of outcomes in NF patients. Second, different classes of antidiabetic treatment can differentially affect glucose levels at admission. In addition, we did not specifically evaluate the effect of glycemic control during hospitalization, although the assumed adequacy of glycemic control during hospitalization may not affect the clinical outcomes or may even have harmful effects [41]. Third, the serum levels of insulin were not routinely measured in NF patients, and we could not evaluate the association between the glycemic gap and insulin resistance. To overcome these limitations, large prospective, randomized, controlled studies are needed to further delineate the relationships among the glycemic gap, SIH and adverse outcomes in diabetic NF patients. Despite these limitations, we believe that the use of the glycemic gap enabled us to explore the predictive value of actual SIH in diabetic patients with NF and that our investigation contains practical information that provides essential insights into areas of future research.

## Conclusions

An elevated glycemic gap and not hyperglycemia at admission is associated with increased incidence rates of NF-related adverse clinical outcomes, particularly bacteremia and AKI, in diabetic NF patients. The glycemic gap may be a useful tool for the prediction of adverse outcomes, and clinicians should consider a more aggressive therapeutic strategy to save lives if the glycemic gap is >146 mg/dL. Further prospective research is needed to confirm this novel finding.

## Supporting information

S1 File Raw clinical data. NF and gap 1080628_PLOS One.
(XLS)
